# Descriptive Study of Prescriptions for Opioids from a Suburban Academic Emergency Department Before New York’s I-STOP Act

**DOI:** 10.5811/westjem.2014.12.22669

**Published:** 2015-01-06

**Authors:** Lyncean Ung, Ronald Dvorkin, Steven Sattler, David Yens

**Affiliations:** * Good Samaritan Hospital Medical Center, Department of Emergency Medicine, West Islip, New York; † Premier Care Physicians, Department of Emergency Medicine, Bellmore, New York; ‡ New York Colleges of Osteopathic Medicine Educational Consortium, New York, New York; § Touro College of Osteopathic Medicine, Middletown, New York

## Abstract

**Introduction:**

Controlled prescription opioid use is perceived as a national problem attributed to all specialties. Our objective was to provide a descriptive analysis of prescriptions written for controlled opioids from a database of emergency department (ED) visits prior to the enactment of the I-STOP law, which requires New York prescribers to consult the Prescription Monitoring Program (PMP) prior to prescribing Schedule II, III, and IV controlled substances for prescriptions of greater than five days duration.

**Methods:**

We conducted a retrospective medical record review of patients 21 years of age and older, who presented to the ED between July 1, 2011 – June 30, 2012 and were given a prescription for a controlled opioid. Our primary purpose was to characterize each prescription as to the type of controlled substance, the quantity dispensed, and the duration of the prescription. We also looked at outliers, those patients who received prescriptions for longer than five days.

**Results:**

A total of 9,502 prescriptions were written for opioids out of a total 63,143 prescriptions for 69,500 adult patients. Twenty-six (0.27%) of the prescriptions for controlled opioids were written for greater than five days. Most prescriptions were for five days or less (99.7%, 95% CI [99.6 to 99.8%]).

**Conclusion:**

The vast majority of opioid prescriptions in our ED prior to the I-STOP legislature were limited to a five-day or less supply. These new regulations were meant to reduce the ED’s contribution to the rise of opioid related morbidity. This study suggests that the emergency physicians’ usual prescribing practices were negligibly limited by the new restrictive regulations. The ED may not be primarily contributing to the increase in opioid-related overdoses and death. The effect of the I-STOP regulation on future prescribing patterns in the ED remains to be determined.

## INTRODUCTION

Pain is one of the most common chief complaints treated in the emergency department (ED), and for many years there has been a strong emphasis on addressing adequate pain control.^1^ Despite the increased importance placed on providing appropriate analgesia, emergency physicians (EP) continue to provide inadequate pain management.^2^ This practice may be related to the clinician’s awareness of the significant problems with drug dependence in the United States. There is an increase in the abuse of prescription drugs, specifically oxycodone and hydrocodone, and it far exceeds the increases in abuse of illicit substances, including marijuana, cocaine, and heroin.^2^

Even with attempts to reduce narcotic abuse and addiction, people continue to exhibit violent behaviors to obtain narcotics. Violent acts led the New York State Legislature to introduce a bill that enhanced the effectiveness of New York’s existing Prescription Monitoring Program (PMP) with the intention of reducing drug diversion.^3^ “The Internet System for Tracking Over-Prescribing (I-STOP) Act” established an online, real-time controlled substance reporting system that mandates physicians and pharmacists to search for and report certain data at the time a schedule II, III, IV, or V controlled substance prescription is issued and at the time such substance is dispensed.”^4^ It took effect in August of 2013. In New York, EPs are waived from the mandatory consultation of the PMP for prescriptions written for five days or less.

There is an association between the maximum prescribed daily dose by any physician and opioid overdose-related deaths, with higher opioid doses related to an increased risk of an opioid overdose death.^5^–^13^ Although multiple studies track the number of prescriptions written by EPs over a period of time, our literature search found no articles that examine the quantity of opioids prescribed to each individual patient during one visit. The duration of pain medications were not included in these particular studies.

We designed this descriptive study to assess the prescription patterns of EPs for opioids prior to the enactment of the New York I-STOP act in a single academic community ED on Long Island. Our expectation is that EPs had been prescribing no more than five days of controlled opioids even before the creation of the new legislation.

## METHODS

This was a retrospective chart review to analyze the prescriptions for controlled opioids prior to the enactment of the I-STOP law. The study was conducted at a suburban academic community hospital ED with an annual census of approximately 95,000 patients. All patients older than 21 years are seen in an adult ED area (69,500).

The database search included all patients 21 and older who received a prescription for a controlled opioid at discharge between the dates of July 1, 2011 and June 30, 2012. We characterized each prescription as to the type of controlled opioid, the quantity dispensed, and the length of time for the prescription. Medical records with insufficient or inconsistent time stamps and/or other entries and patients who left prior to formal discharge were excluded. The data extracted included the following: hospital account number, age, sex, ethnicity, diagnosis, arrival date, time, and prescriptions written.

We abstracted the data from the electronic medical record (Allscripts ED™ - formerly Healthmatics A4™) for patients discharged from the ED with one or more prescriptions. This computerized patient charting and order entry system enables the collection of standardized information for each patient and integrates that information into a relational database and also has the advantage of generating prescriptions that were included in the medical record. The database was queried by SQL Cognos Impromptu™ (Cognos), which allows the administrator to create reports using criteria filters. Using the inclusion criteria, a report was created with Cognos that queried all patients seen in the ED who fit the criteria for study enrollment. This report was further analyzed by Microsoft Excel 2007. This method has been used and described in other studies.^14^–^16^

The prescriptions analyzed were those written for opioids including hydrocodone, morphine, hydromorphone, and fentanyl (patches) as seen in Table 1. Tramadol was not analyzed, as it only has been considered a controlled substance in New York since August 27, 2012. We also excluded a cough syrup containing hydrocodone with homatropine in low dose (Hycodan^®^) because it was never prescribed in amounts of more than 120mL, which was equivalent to the total hydrocodone of 24 pills (a quantity usually of no more than five days as traditionally prescribed), and we could find little evidence for this significantly contributing to the epidemic of prescription drug abuse overdoses or deaths.

**Figure 1:**
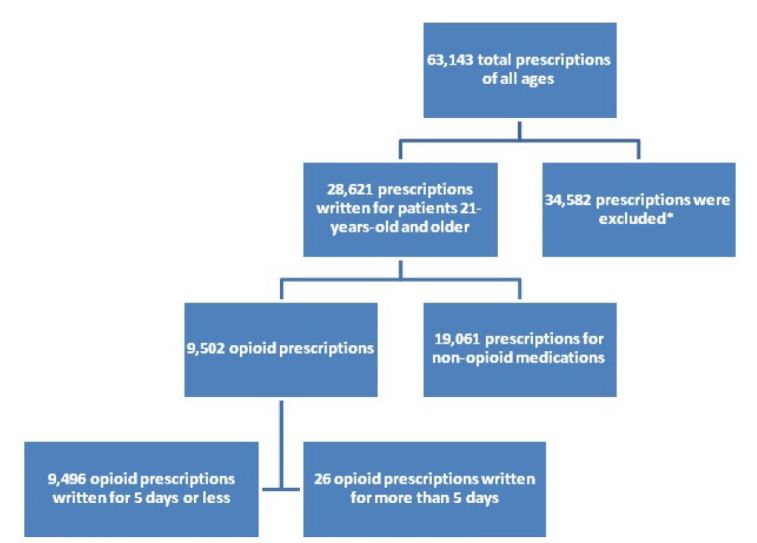
Analysis of prescriptions written in the emergency department. ^*^The prescriptions that were excluded as listed in the methods section include those written for patients who are less than 21 or have a malignancy and chronic inflammatory disease. Patients who also received Hycodan^®^ were also excluded.

We calculated the number of days prescribed by using the number of doses prescribed, shortest recommended dosing frequency and converting this to the number of days the prescription would last (Figure 1). All patient charts that had a prescription of more than five days were manually reviewed.

## RESULTS

**Figure 2:**
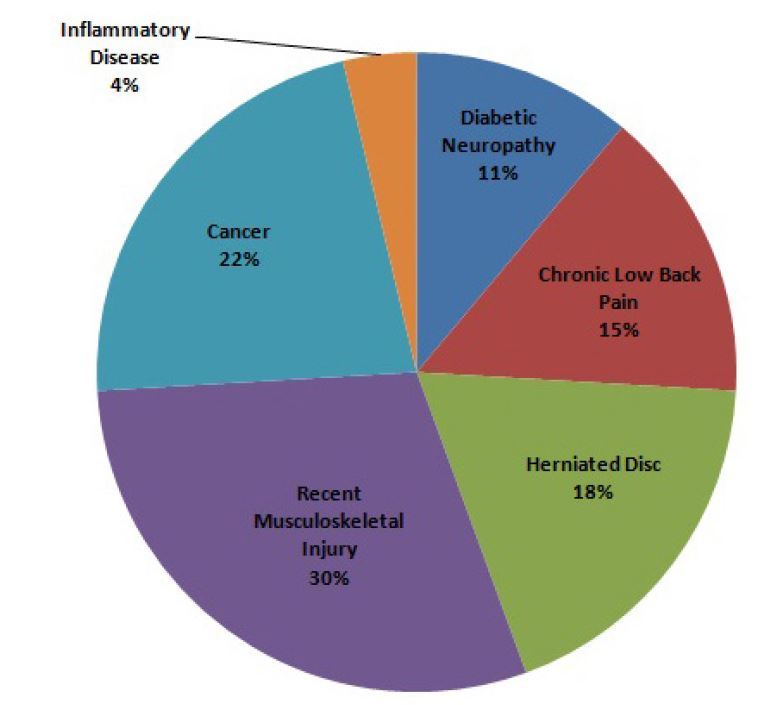
Diagnosis of patients receiving opioid prescriptions for greater than 5 days (n=26).

**Figure 3:**
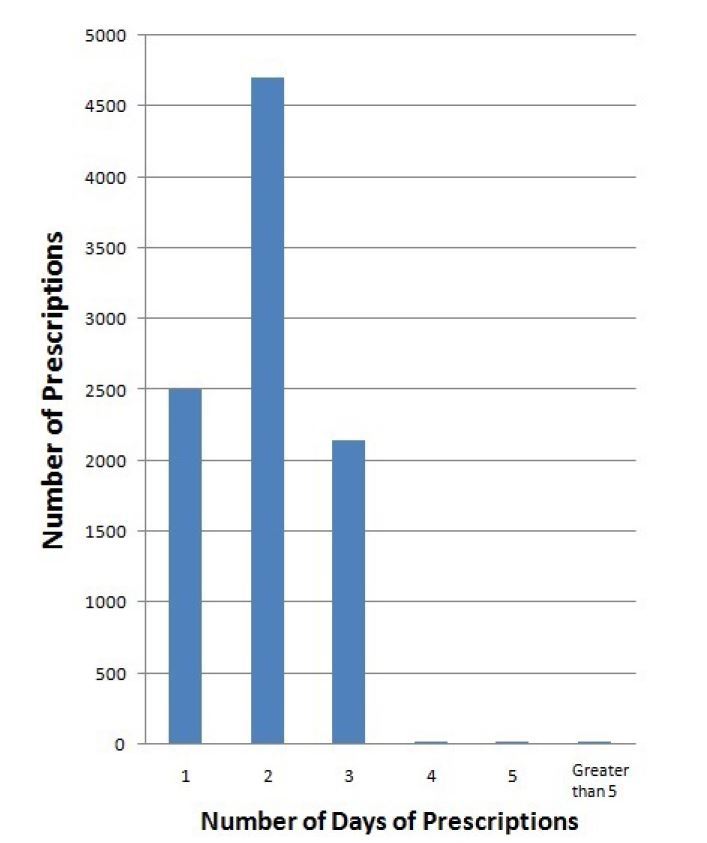
Breadkdown of opioid prescriptions into the number of days they were written for.

A total of 63,143 prescriptions were written between July 1, 2011 and June 30, 2012. The demographics are listed in Table 2. Of those prescriptions, 9,502 prescriptions were written for opioids. We excluded 71 prescriptions from the study because they were duplicates of the original prescription or lacking information on the prescription to calculate the number of days. Of the 9,502 prescriptions written that were included in our study, 99.7% already complied with the new regulations (95% CI [99.6 to 99.8%]). Twenty-six prescriptions (0.27%) were written for greater than five days as seen in Figure 2. Some were a result of larger intervals between each dose than typically recommended. For example, fentanyl usually is packaged in a box of five patches and each patch is used for three days at a time. The prescriptions written for greater than five days were categorized by diagnosis. A breakdown of the 26 outlying prescriptions and related diagnoses are provided in Figure 2 with their corresponding diagnoses. All written opioid prescriptions were further divided by the number of days they were written for, which is seen in Figure 3.

Our study found that prescriptions for opioids were usually for no more than five days in length and would have almost always complied with the new state regulations.

## DISCUSSION

I-STOP made New York the first state in the nation to mandate that physicians consult a database of a patient’s prescription history before prescribing a schedule II, III, or IV controlled substance.^17^ Studies analyzing the impact of PMPs have been unanimous in regards to decreasing drug dependence and diversion by inhibiting growth in prescription sales for pain relievers. This act may further enhance the PMP’s goal; however, it can be hindering in an emergency setting. There is ongoing concern about EPs undertreating pain and the majority of emergency patients suffering from acute injury.

In the ED, pain management continues to be a significant complaint requiring practitioner attention as seen from the data of this study where approximately one-sixth of all prescriptions written were for opioid analgesics. As seen in numerous studies conducted previously, oxycodone continues to be the most commonly prescribed opioid (84%).^15^,^18^–^20^ We were able to illustrate that in a large suburban, community ED, most physicians were prescribing five days or less worth of prescriptions for opioids, with the majority being for three days or less. This pattern could be affected by the presence of an electronic medical record (EMR) that has prebuilt prescriptions for a set number of pills. Even though prescriptions could be typed up individually with varying dosing and number of pills, it might be more efficient for a physician to select a prebuilt prescription.

Of the opioids considered in this study, short-acting oxycodone is still the most commonly prescribed opioid as well as the most commonly prescribed for greater than five days. The small number of patients who received more than five days’ worth of opioids had pain from acute musculoskeletal injuries, chronic low back pain, herniated disc, and diabetic neuropathy.

Giving ED physicians the privilege of providing opioid pain relievers without referring to the PMP permits them to function effectively in their environment. Our study shows that the EP already displays independent responsibility in opioid prescribing patterns. The PMP in theory may add little to reduce the amount of drug overdoses in the ED setting.

Prior to enactment of this legislation, EPs had not been giving prescriptions of opioids that were excessive and therefore not likely to be contributing to the rise of opioid overdoses and deaths in the community. Further regulations requiring the additional step of consulting a database before prescribing controlled opioids may have the potential to inhibit opioid prescriptions for acute pain and promulgate oligoanalgesia.

## LIMITATIONS

Our study has several limitations. It was a retrospective study and has all the limitation therein. It was completed at a single suburban academic community hospital ED and therefore might not represent other communities or institutions. It cannot determine if the patient is “doctor-shopping” and visiting other EDs, doctors’ offices, or even the same ED but on subsequent days or weeks. We focused on the duration of opioid prescriptions written rather than the maximum concentration of the opioid all together or total per day. There is increased risk of overdose in patients receiving medically prescribed opioids at higher doses.^5^–^13^

Patients can still “doctor-shop,” and if an EP does not have a clinical suspicion for substance abuse, the small amounts of opioids given can supply an addiction from multiple sources. Furthermore, a patient’s inability to obtain a sufficient quantity of prescription opioids may lead them to solicit street drugs that have unregulated compositions adding to potentially worse adverse outcomes.

The study did not provide any data on the type of pain or response to initial treatment. It was not designed to determine whether pain was adequately treated. The results could be influenced by variations in individual provider practice, and the study did not control for the effect of clustering by individual providers. Providers in this department see patients on a next patient basis and do not choose the next patient to be seen, thus minimizing this type of selection bias.

Whether the implementation of this regulation will change the prescribing practices of physicians is not known and would be an interesting question to pursue.

## CONCLUSION

The duration of opioids prescribed from one ED visit in this suburban, community Long Island ED prior to the I-STOP legislature was largely limited to five days or less. This suggests that EPs had been largely abiding with the spirit of the new bill prior to its drafting. Although the PMP might assist with abuse and dependency of opioids in this community, it remains unknown whether this will impact optimal prescribing practices for pain.

## Figures and Tables

**Table 1:** Percentage of opioid prescriptions written.

Opioids (n)	% (No.) of prescriptions
Oxycodone	84.08% (8040)
Hydrocodone	14.47% (1324)
Morphine	0.02% (2)
Hydromorphone	1.24% (119)
Fentanyl	0.18% (17)

**Table 2:** Demographics of patients receiving opioid versus non-opioid prescriptions.

	Opioid	All prescription
N	9,502	28,621
Mean age, y (SD)	44.9 (15.2)	44 (16.4)
Male (%)	42.8%	43.2%
White (%)	61.2%	61.5%
